# SIG-Former: monocular surgical instruction generation with transformers

**DOI:** 10.1007/s11548-022-02718-9

**Published:** 2022-07-28

**Authors:** Jinglu Zhang, Yinyu Nie, Jian Chang, Jian Jun Zhang

**Affiliations:** 1 National Centre for Computer Animation, Bournemouth University, Bournemouth, UK; 2 Technical University of Munich, Munich, Germany

**Keywords:** Surgical instruction generation, Transformer, Image captioning, Reinforcement learning

## Abstract

**Purpose:**

Automatic surgical instruction generation is a crucial part for intra-operative surgical assistance. However, understanding and translating surgical activities into human-like sentences are particularly challenging due to the complexity of surgical environment and the modal gap between images and natural languages. To this end, we introduce **SIG-Former**, a transformer-backboned generation network to predict surgical instructions from monocular RGB images.

**Methods:**

Taking a surgical image as input, we first extract its visual attentive feature map with a fine-tuned ResNet-101 model, followed by transformer attention blocks to correspondingly model its visual representation, text embedding and visual–textual relational feature. To tackle the loss-metric inconsistency between training and inference in sequence generation, we additionally apply a self-critical reinforcement learning approach to directly optimize the CIDEr score after regular training.

**Results:**

We validate our proposed method on DAISI dataset, which contains 290 clinical procedures from diverse medical subjects. Extensive experiments demonstrate that our method outperforms the baselines and achieves promising performance on both quantitative and qualitative evaluations.

**Conclusion:**

Our experiments demonstrate that SIG-Former is capable of mapping dependencies between visual feature and textual information. Besides, surgical instruction generation is still at its preliminary stage. Future works include collecting large clinical dataset, annotating more reference instructions and preparing pre-trained models on medical images.

## Introduction

With the increasing demands of surgical training, intra-operative surgical assistance and decision support in modern clinical rooms, digital context-aware surgical system is an essential component toward next-generation surgery. It aims to leverage the available information inside operation rooms to assist clinicians during surgical practices. Among all the related techniques, surgical instruction generation is a process of generating human-like guidance from surgical views. It is particularly important when an emergency situation is detected or onsite mentoring is unavailable. However, the complexity of operation environments, the high intra-procedure variance and low inter-procedure variance make this task particularly challenging.

Previously, telementoring [4] is an alternative solution by utilizing telecommunication techniques to provide remote surgical guidance and technical assistance. But this technique highly hinges on the quality of telecommunication systems and is always influenced by some legal and ethical issues [2, 10]. More recently, transformative technologies in computer-aided surgery bring potentials to understand the surgical activities and provide context-aware assistance from different perspectives. For example,  [8, 11, 22, 27] apply vision-based deep learning methods for surgical workflow analysis and fine-grained surgical gesture recognition. However, these techniques depend on pre-defined surgical phases and gesture classes, which are incapable of understanding the holistic surgical view and generating human-like instruction.

Medical report generation [5, 6, 13] is the closest topic to our task, which automatically generates diagnostic report for a patient with text descriptions and lists of tags from radiology and pathology images. Nonetheless, medical report generation follows some templates, for instance, it always includes few fixed sentence templates where each one focuses on one specific topic. While in surgical instruction generation task, the surgical content and the way describing it are both diverse.

To the best of our knowledge, the only prior work of surgical instruction generation task is [20]. In their study, the authors collect a dataset called Database for AI Surgical Instruction dataset (DAISI) and use bidirectional recurrent neural network (RNN) method to build a baseline model. This work, however, has two main limitations. On the one hand, although the RNNs are designed to memorize the historical information and generate sequences in arbitrary length, they have limited representation ability and are bottlenecked by the gradient vanishing and exploding problems [17]. On the other hand, evaluating the quality of sentences in different points of view is significant to verify their correctness. Nevertheless, they use the BLEU score [16] as the only evaluation metrics, which is deficient.

**Fig. 1:**
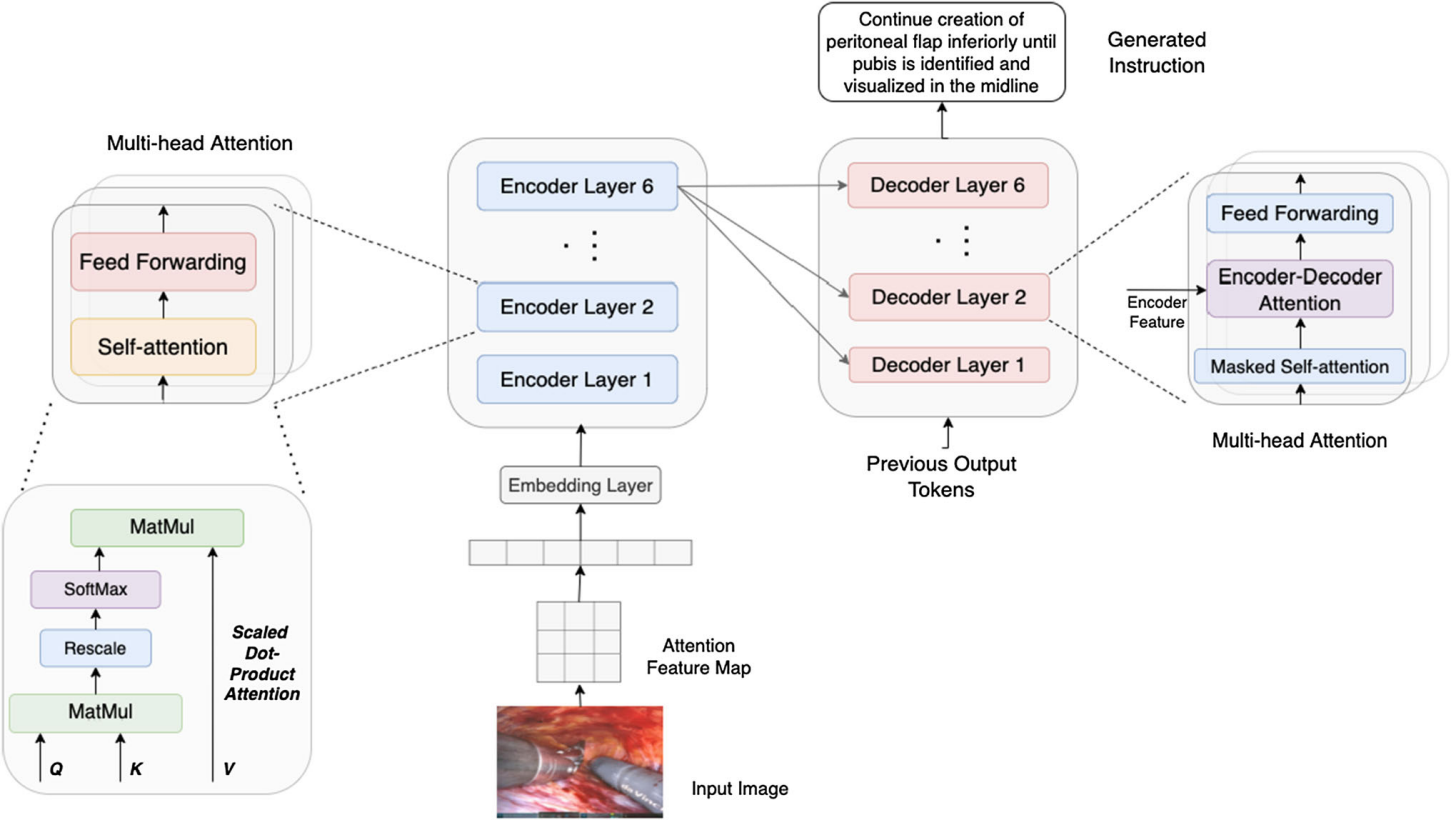
The architecture of our SIG-Former model

In this paper, we propose **SIG-Former**, a transformer-backboned encoder–decoder architecture along with self-critical reinforcement optimization, to generate instruction texts from surgical images. Our proposed methods are mainly based on two insights and observations: (1) With self-attention and multi-head attention mechanism, transformers can achieve great performance in sequence generation, for example, machine translation and image captioning from natural domain [7, 23]. (2) Sequence generation tasks are often trained with the teacher forcing mode [18]. That means during the training procedure, the model uses the ground-truth token to predict the next token, while it uses the previous generated token to predict the next token during the inference stage. This discrepancy between training and inference procedure causes accumulated errors.

Given a surgical image, we first extract its attention map using a pre-trained ResNet-101 model [12]. Then we feed the attention map into a transformer encoder to get the position-wise latent feature map. The transformer encoder–decoder attention module and decoder attention module further models the visual-text dependency and token-wise textual information. Furthermore, after the standard cross-entropy training for some epochs, we employ the self-critical reinforcement learning to alleviate aforementioned mismatching between training and inference.

We validate the effectiveness of our approach on DAISI dataset. This work is an extended version from MICCAI2021 conference paper [27], where we improve our training strategy by exploring the data distribution. The results demonstrate that our new training setup further improves the performance of SIG-Former in surgical instruction generation.

## Methods

The architecture of SIG-Former is shown in Fig. 1. It consists of two parts: (1) surgical instruction generation with a transformer-based encoder–decoder model (see Sect. 2.1) and (2) self-critical reinforcement learning (see Sect. 2.2).

### Surgical instruction generation with transformers

Rather than computing the tokens sequentially in recurrent style, transformer [23] allows building non-local relationships concurrently for different positions inside a sequence. Our SIG-Former model has two components: a transformer encoder and a transformer decoder, with each composed of stacks of attentive layers. The encoder input is feature maps from surgical images while the output is a sequence of tokens (i.e., the surgical instructions).

Given a surgical image, we first extract its feature map, with the dimension of $14\times 14\times 2048$, using the last convolutional layer of a pre-trained ResNet-101 [12]. We reduce the feature map to $14\times 14\times 512$ dimensions with a linear embedding layer followed by a ReLU activation layer and a dropout layer. We further flatten the feature maps to the shape of $196\times 512$ and put this visual embedding as the entry token to the first transformer encoder layer.

The transformer encoder aims to build position-wise relationships for input image regions. It is composed of a sequence of six identical attention modules, where each module consists of a multi-head self-attention layer followed by a feed-forward layer. For a given input $X\in R^{N\times D}$, where *N* is the number of entries and *D* is the feature dimension, the attention layer first linearly converts the input into queries ($Q= XW_Q, W_Q\in R^{D\times D_k}$), keys ($K = XW_K, W_K\in R^{D\times D_k}$) and values($V = XW_V, W_V\in R^{D\times D_v}$). Then the scaled-product attention can be computed by:

1 $$\begin{aligned} \mathrm {Attention} (Q,K,V) = \mathrm {Softmax} \left( \frac{QK^T}{\sqrt{D_k}}\right) V \end{aligned}$$

where $D_k$ is the dimension of queries and keys; $D_v$ is the dimension of values ($D_k$ = $D_v$ in our implementation). In order to jointly access to different sub-spaces, the **Multi-head attention** is applied (#heads = 8). The outputs from 8 heads are then concatenated and multiplied by a learned projection matrix $W_O$. The process can be represented as:

2 $$\begin{aligned} \begin{aligned} \mathrm {MultiHead}(Q,K,V)&= \mathrm {Concat}(\mathrm {head}_1,\ldots ,\mathrm {head}_h)W_O\\ head_{i}&= \mathrm {Attention}(X{W_{Q_i}}, X{W_{K_i}}, X{W_{V_i}}). \end{aligned} \end{aligned}$$

The next component applied to the output of each attention layer is a position-wise feed-forward layer:

3 $$\begin{aligned} \mathrm {FFN(x)} = \mathrm {max}(0,xW_1 + b_1)W_2 + b_2, \end{aligned}$$

where $W_1$, $b_1$ and $W_2$, $b_2$ are the learnable weights and biases of two MLP layers.

The transformer decoder is also a sequential stack of six identical attention modules, where each module has two layers of multi-head attention (one for the self-attention on words and another for cross attention over the output from the last encoder layer) followed by one position-wise feed-forward layer. For the detailed explanation of the decoder, we refer readers to [23].

### Self-critical reinforcement optimization

Sequence generation models usually come with two shortcomings: (1) During the training process, the model predicts next word using the previous ground-truth word. While in inference, the model predicts the next word by feeding the previous generated word as the input. This discrepancy is called *exposure bias* [18] because the model is only exposed to the training data distribution rather than its own prediction. As the result, the error would be quickly accumulated if initial predictions are wrong. (2) Another mismatching is in the loss calculation. In the training stage, normally the word-level cross-entropy loss is used to maximize the likelihood of the next correct word. However, the non-differentiable language evaluation metrics (e.g., BLEU or CIDEr) are applied to evaluate the model performance.

**Fig. 2:**
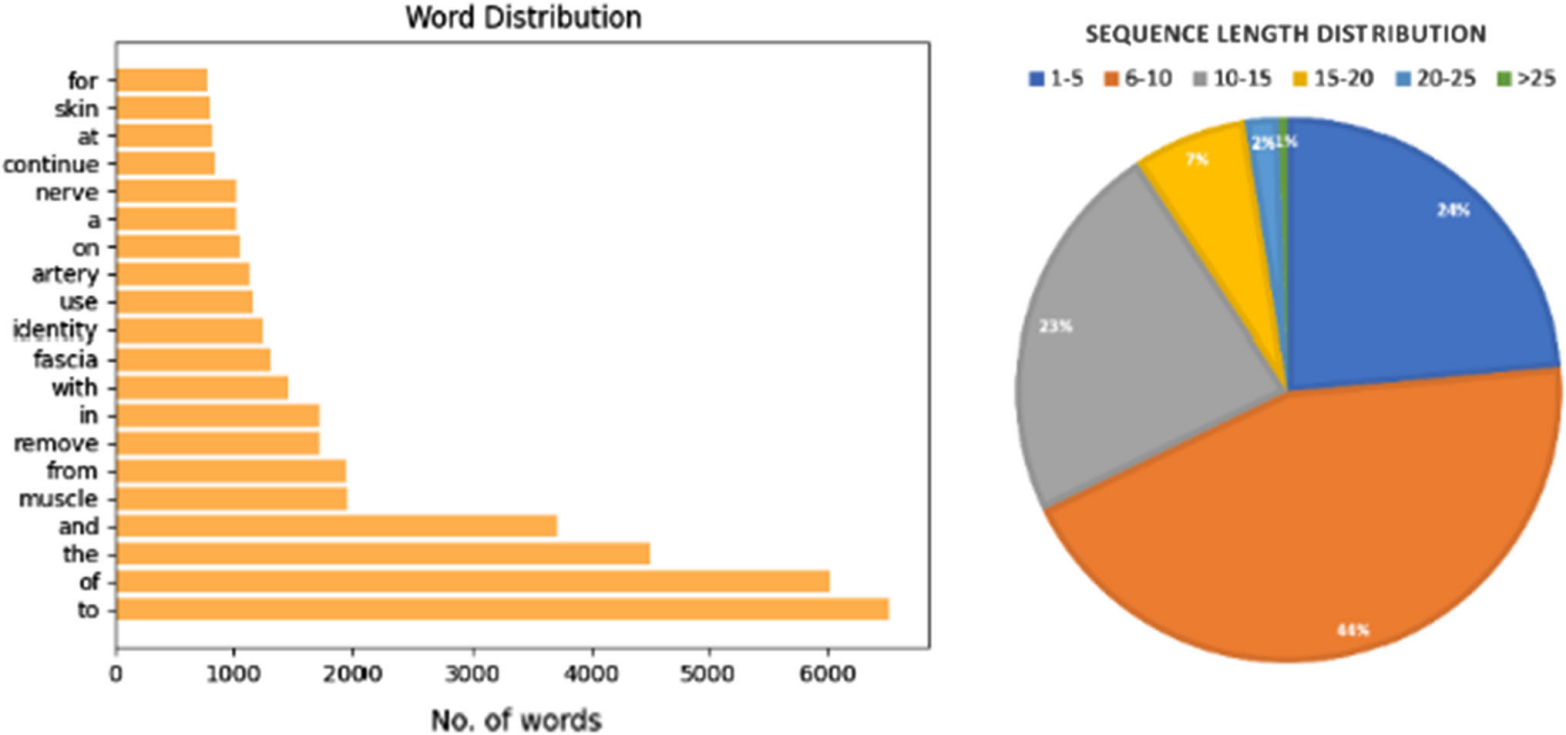
Top-20 words distribution and sequence length distribution

In order to eliminate above discrepancies, following the standard practice in image captioning from natural domain, we first train our model with word-level cross-entropy and then fine-tune the sequence generation model using self-critical reinforcement learning approach. Consequently, in the reinforcement fine-tuning step, we use predicted words as the input to generate the next word in training and directly optimize the model with CIDEr score as the reward. Because it well correlates to human judgement [24].

Following the details from [19], given the model parameters $\theta$, the policy $p_\theta$ and sentence $w^\mathrm{s}$, the gradient for one sample can be expressed as:

4 $$\begin{aligned} \nabla _{\theta }L(\theta ) = -\mathbb {E}_{{w^\mathrm{s}}\sim p_\theta }[(r(w^\mathrm{s})-b)\nabla _{\theta }\log p_{\theta }(w^\mathrm{s})] \end{aligned}$$

where *r*(.) is the reward function, and $b=r{\hat{w}}$ is the self-critical baseline, which is obtained by the current model under the inference algorithm applied at the test time. As the result, it increases the probability of high reward sample and penalties the low reward sample. For detailed formula derivation, please refer to [19].

## Results and evaluation

### Experimental details

**Dataset.** We evaluate our proposed method on the DAISI dataset [20]. The dataset contains 17,256 color images of the 290 medical procedures from 20 clinical disciplines, including laparoscopic inguinal hernia repair, open cricothyroidotomy, laparoscopic sleeve gastrectomy, etc. The availability of the dataset is upon request from.^1^ Each procedure contains surgical images with their corresponding text description of how to complete a step in the procedure. We further clean the dataset by deleting the irrelevant and noisy images and captions, e.g., some images and captions only represent the surgeon information of that particular procedure. Finally, we get 16,413 images with one caption per image. Rather than split the images randomly in [27], we split the data intra-procedurally. That means inside each procedure, we randomly assign 80% images for training, 10% for validation and 10% for testing. We finally have 13,035 training images, 1618 validation images and 1760 test images.

**Text Preprocessing.** Text preprocessing is a significant task for any natural language-related task. The unstructured raw text data need to be converted to a more digestible and predictable format such that the model can learn meaningful feature and perform better. We follow these four steps to clean the raw text data: (1) Convert all the words into lower case; (2) Expanding abbreviations, including English contractions (e.g., “aren’t” to “are not”) and medical abbreviations (e.g., “m.” to “muscle”); (3) Remove all numbers, whitespaces and punctuation; (4) Tokenize the sentence into words. Moreover, we set the sentence length threshold to 16 and mark those words as “UNK” if they appear less than 5 times in dataset, which ends up with a 2212 words vocabulary. The top-20 word distribution and sentence length distribution are shown in Fig. 2.

**Evaluation Metrics.** Automatically evaluating the quality of text descriptions is important as human-based evaluation is unaffordable. Following the standard evaluation protocol, we apply the common metrics for evaluation, i.e., accumulated 1-4 BLEU [16], Rouge-L [14], METEOR [3], CIDEr [24] and SPICE [1].

**Table 1:** Comparison with the state of the arts [20] for surgical instruction generation task

Surgical instruction	*B*1	*B*2	*B*3	*B*4	*C*	*M*	*R*	*S*
DAISI (Bi-RNN)	21.0	14.4	11.3	9.3	8.32	10.3	22.0	12.1
LSTM [27]	43.7	39.4	37.3	36.2	34.0	24.9	44.6	40.2
Soft-attn [27]	43.2	38.7	36.3	34.9	32.4	24.3	43.7	38.0
Transformer only [27]	45.5	41.0	38.7	37.2	34.0	25.6	44.3	39.7
Transformer + rl (ours) [27]	52.8	48.7	46.4	44.9	42.7	30.7	53.1	48.4
LSTM	42.0	37.1	34.6	33.0	30.63	24.0	41.9	36.2
Soft-attn	44.6	39.7	37.1	35.5	32.79	24.8	44.9	38.9
Transformer only	52.4	48.0	45.5	43.8	41.32	29.9	52.8	46.7
Transformer + rl (ours)	56.1	52.2	49.8	48.2	45.61	32.7	56.6	50.9

B1, B2, B3, B4, C, M, R and S stand for 1–4 gram BLEU, CIDEr, METEOR, ROUGE-L and SPICE score, respectively. The upper and the lower part of this table present the evaluations on random data split and the intra-procedure data split, respectively. rl indicates reinforcement learning

### Implementation details

To comprehensively explore the performance of SIG-Former in surgical instruction generation, we additionally implement two LSTM-based generation models as baselines. We implement all methods using PyTorch and train them on two GeForce RTX 2080 Ti GPUs.

**Our method.** We extract the input feature map with the last convolutional layer of a pre-trained ResNet-101 [12], which is followed by a spatial adaptive max-pooling layer and flattened to $196 \times 2048$ dimension. For the standard cross-entropy training, we set the batch size to 16. The learning rate of the model is initialized to $3\times 10^{-4}$ and follows the learning rate scheduling strategy with 20,000 warm-up steps. After 50 epochs training with cross-entropy loss, we employ the self-critical reinforcement strategy to optimize the CIDEr score with the fixed learning rate of $1\times 10^{-5}$ for another 10 epochs. The batch size for this stage is set to five.

**LSTM-based methods.** We further provide two LSTM-based baselines for the surgical instruction task (LSTM and LSTM-based soft-attention model) similar to [25, 26], as this task is fairly new and LSTM is a milestone work for processing sequences. We also use the last convolutional layer of a pre-trained ResNet-101 to extract visual features for these two baselines. We apply an average pooling and obtain 2048-d feature for vanilla LSTM. It is trained with the initial learning rate to $5\times 10^{-4}$ and batch size to 16 for 70 epochs using cross-entropy loss.

The LSTM-based soft-attention model shares the same feature map ($196 \times 2048$ dimension) with our SIG-Former model. For the cross-entropy training stage, we initialize the learning rate to $5\times 10^{-4}$ and batch size to 16. For the reinforcement optimization step, the learning rate is fixed to $1\times 10^{-5}$ with batch size at five.

All the models are optimized using the ADAM optimizer.

### Comparison with the state of the arts

Since we clean the data by removing noisy and inappropriate image-text pairs, a new evaluation benchmark is required. We re-implement the state-of-the-art network Bi-RNN [20] for comparison, as their code is not publicly available. Following the details of Bi-RNN, we extract the 4096-d feature using the last convolutional layer of a pre-trained VGG16 network [21]. The Bi-RNN model is trained 50 epochs with learning rate at $5\times 10^{-4}$ and batch size at 10.

Rather than randomly split the data as in [27], we split the data *intra-procedurally* such that the model is able to get some prior information during the test stage (see Sect. 3.1). We compare the performance of our method with the state-of-the-art and other baseline methods in Table 1. To verify the efficacy of reinforcement learning, we also ablate our method by removing it in evaluation, which is denoted by “Transformer only” in Table 1.

It can be seen that Bi-RNN method shows a relatively lower performance than other proposed baselines on all the evaluation metrics. For example, CIDEr [24] is an evaluation metric specifically designed for image captioning task based on the consensus between predicted instructions and reference descriptions. The CIDEr score of Bi-RNN is 30% lower than ours, which indicates the weakness of a simple RNN model in catching visual–textual relationship for instruction generation. For other three proposed approaches, transformer model with reinforcement learning outperforms all other methods on all evaluation metrics. The promising results of transformer models indicate the robustness of the encoder–decoder attentive layers.

From another perspective, one intuitive difference between natural domain image captioning and surgical instruction generation is that there are strong contextual relationship and temporal dependency between images in the same type of surgical operations, which is particularly important for surgical content analysis. In Table 1, we see the *intra-procedure* split further improves the model performance, where the scores of transformer-only model show $\approx 7$ points higher than its performance in random split. Since for the *intra-procedure* setting, 80% of the images in the same procedure are assigned to the training set and the rest are in the validation and test set, each model is equipped with more prior information from the training set.

**Fig. 3:**
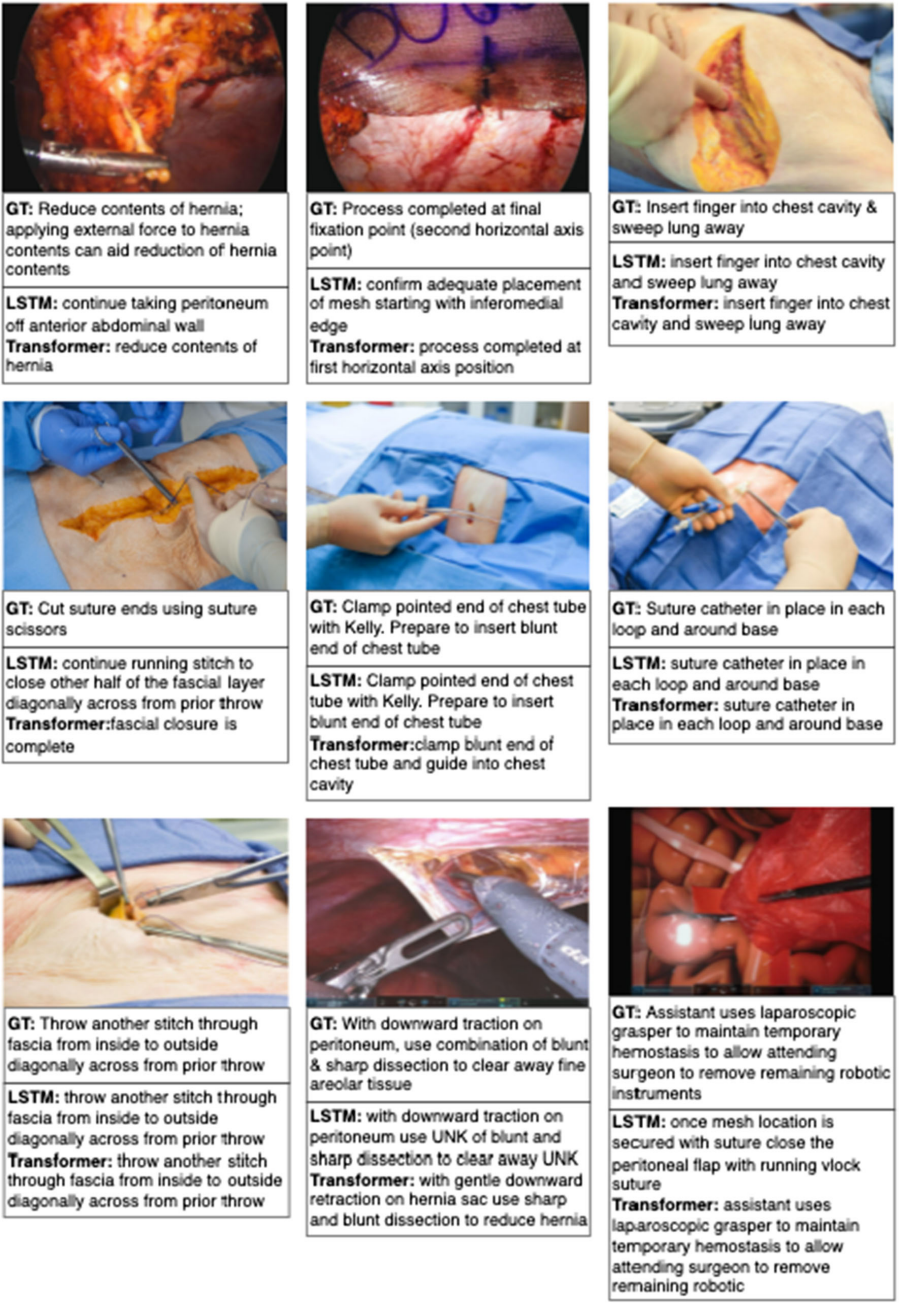
Qualitative evaluation for SIG-Former. We randomly select 9 images with predicted instructions from LSTM and SIG-Former

Figure 3 shows the qualitative comparisons. We randomly select 9 images with predicted instructions from the best LSTM model and our SIG-Former.

**Table 2:** Ablative study to understand the functionality of each module

Surgical instruction	*B*1	*B*2	*B*3	*B*4	*C*	*M*	*R*	*S*
LSTM	42.0	37.1	34.6	33.0	30.63	24.0	41.9	36.2
Soft-attn	44.6	39.7	37.1	35.5	32.79	24.8	44.9	38.9
Soft-attn + rl	44.9	39.7	37.3	35.4	33.06	24.8	45.2	39.3
Transformer only	52.4	48.0	45.5	43.8	41.32	29.9	52.8	46.7
Transformer + rl (ours)	56.1	52.2	49.8	48.2	45.61	32.7	56.6	50.9

B1, B2, B3, B4, C, M, R and S stand for 1–4 gram BLEU, CIDEr, METEOR, ROUGE-L and SPICE score, respectively

## Discussion

### Ablation study

To explore the effectiveness of each single design in our method, we decompose our network into six configurations as follows:

***C1:*** LSTM only
***C2:*** Soft-attention only
***C3:*** Transformer only
***C4:*** Soft-attention + Self-critical reinforcement learning
***C5:*** Transformer + Self-critical reinforcement learning

The results are presented in Table 2, from which we observe that:

***C1 versus C2***: We add the soft-attention on top of the LSTM model such that each word position can sequentially access different image regions to make prediction.

***C1 versus C2*** and ***C3***: Without using any sequence-aligned recurrent units, the transformer attention mechanism processes the sequence as a whole. Comparing with other baselines, transformer backboned framework improves the performance over all evaluation metrics, which demonstrates the capability of transformers in processing multi-modal context.

***C2 versus C4***: and ***C3 versus C5***: After the standard training stage with cross-entropy loss, we use the reinforcement learning to directly optimize the CIDEr score. From Table 2, we see that this optimization step not only improves the CIDEr score, but also improves the results on other metrics. Specifically, there is an obvious improvement for the transformer-only model.

### Limitations and challenges

In this section, we discuss the limitations and challenges in the surgical instruction generation task.

1. Limited dataset scale. Surgical instruction generation is a multi-modal task which relates to visual, textual and dependencies between them, where the parameter space is much larger than those single-modal tasks (e.g., classification or objection detection). It requires large amount of data to tune complex hyper-parameters and prevent overfitting. In natural image domain, the COCO image captioning task [15] has more than 120k samples, while the DAISI dataset has less than 20K images. Furthermore, from the comparisons in Table 1, contextual information contributes to the performance of the models. However, the dataset contains only one sample for few surgical procedures, thus only spatial-based prior information can be learned. If we can build a larger dataset, not only the spatial information but also the contextual temporal relationships can be learned to improve the instruction generation.
2. No fine-grained supervisions. In natural image domain, a large dataset (e.g., COCO or ImageNet [9]) is usually pre-trained to support downstream tasks such as object detection and attribute feature identification. Nonetheless, these pre-trained models are difficult to be applied in medical domain as labeling surgical images requires expert annotators.
3. One reference instruction per image. In real case, the content for a same image can be explained in multiple ways. COCO image captioning dataset [15] equips one image with 5 different references, while we have only one in the DAISI dataset, where an appropriate prediction could be ignored only because it has a different instruction description.

## Conclusion

In this paper, we propose a self-critical transformer, named by SIG-Former, to generate surgical instructions given from a monocular image. The network is composed of a transformer encoder to model visual features, a transformer decoder to model textual information and an encoder–decoder to catch multi-model dependencies. In addition, we use the reinforcement learning approach to alleviate the discrepancy between training and inference by directly optimizing the CIDEr metric. The performance of our method demonstrates the effectiveness of attention blocks in handling multi-modal sequence-to-sequence problem.

## Footnotes

1 https://engineering.purdue.edu/starproj/.
